# The S‐Phase Arrest of Host Cells Caused by an Alpha‐Herpesvirus Genome Replication Facilitates Viral Recruitment of RNA Polymerase II to Transcribe Viral Genes

**DOI:** 10.1111/cpr.13811

**Published:** 2025-01-27

**Authors:** Qiqi Yang, Ying Wu, Mingshu Wang, Shun Chen, Renyong Jia, Qiao Yang, Dekang Zhu, Mafeng Liu, Xinxin Zhao, Shaqiu Zhang, Juan Huang, Xumin Ou, Di Sun, Bin Tian, Yu He, Zhen Wu, Anchun Cheng

**Affiliations:** ^1^ Engineering Research Center of Southwest Animal Disease Prevention and Control Technology Ministry of Education of the People's Republic of China Chengdu China; ^2^ International Joint Research Center for Animal Disease Prevention and Control of Sichuan Province Chengdu China; ^3^ Key Laboratory of Animal Disease and Human Health of Sichuan Province Sichuan Agricultural University Wenjiang China; ^4^ Avian Disease Research Center College of Veterinary Medicine of Sichuan Agricultural University Wenjiang China

**Keywords:** AnHV‐1, cell cycle, gene transcription, genome replication, RNA polymerase II

## Abstract

Herpesviruses rely on host RNA polymerae II (RNA Pol II) for their mRNA transcription, yet the mechanisms of which has been poorly defined, while certain herpesviruses can enhance viral gene transcription by altering the RNA Pol II location, modulating its phosphorylation, or directly interacting with RNA Pol II. However, the influence of herpesviruses on RNA Pol II transcription extends beyond these direct effects. Here, we present a novel mechanism by which the host cell cycle regulates viral gene transcription via RNA Pol II during infection by Anatid Herpesvirus 1 (AnHV‐1), an avian alpha‐herpesvirus. The results demonstrated that the formation of viral replication compartments (vRCs) and the subsequent recruitment of RNA pol II are positively correlated with AnHV‐1 DNA synthesis. As viral DNA replication progresses, host cells are arrested in the S phase, which not only halts host gene transcription but also facilitates viral transcription. This cell cycle arrest in the S phase promotes viral DNA (vDNA) synthesis and vRC formation, which further enhances the preferential recruitment of RNA Pol II to viral promoters, enabling efficient viral gene transcription. We propose that this S phase arrest and the hijacking of RNA Pol II represent a novel mechanism by which AnHV‐1 enhances viral transcription, offering a unique survival strategy compared to the known strategy in herpesviruses. These findings expand our understanding of herpesvirus–host interactions and highlight potential targets for antiviral strategies.

## Graphical abstract

Viral infection arrests host cells in the S phase, which promotes vDNA synthesis and vRC formation, further facilitating viral recruitment of RNA Pol II to preferentially transcribe viral genes.

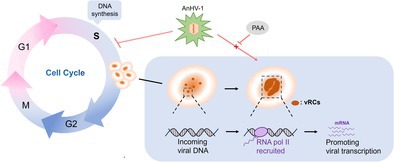

## 1 Introduction

Viruses rely on infected cells for resources throughout their life cycle. To replicate efficiently, viruses have evolved multiple mechanisms to manipulate the environment of infected cells to compete for resources [1, 2, 3]. One critical strategy used by many viruses is hijacking the host's transcription machinery to transcribe viral genes, allowing the viruses to compete for cellular resources. However, the mechanism underlying the recruitment of RNA pol II by viruses remains poorly understood. Current research on RNA Pol II recruitment by herpesviruses largely focuses on the direct manipulation of the polymerase by viral proteins, without fully addressing how these processes are linked to the cell cycle. For example, herpes simplex virus 1 (HSV‐1) shuts down host gene expression and promotes viral transcription by altering RNA pol II localisation and inhibiting RNA pol II S2 phosphorylation [4, 5, 6, 7]. HSV‐1 ICP27 could bind to RNA pol II CTD and direct to the replication site [8], and ICP22 and UL13 mediated the phosphorylation of RNA pol II [9, 10]. HSV‐1 ICP22 and UL36 are able to prevent ubiquitination degradation of the phosphorylated form of RNA pol II S2 [11]. Similarly, the human cytomegalovirus (HCMV) UL79 and Kaposi's sarcoma‐associated herpesvirus (KSHV) ORF24 are able to recruit RNA pol II to promote viral gene transcription [12, 13]. KSHV LANA is also able to inhibit RNA pol II degradation [14]. Additional Epstein–Barr virus (EBV) EBNA 2 stimulates both pol II recruitment and pol II phosphorylation on serine 5 of the CTD in vivo [15]. Despite these insights, the relationship between viral‐induced cell cycle arrest and RNA Pol II hijacking is not well understood.

Notably, several herpesviruses are known to manipulate the host cell cycle to optimise conditions for viral replication. For instance, the three representative herpesviruses HSV‐1, HCMV, and EBV arrest the G1/S transition of the cell cycle, a strategy that allows the virus to bypass the S phase, blocks cellular DNA replication, and avoids competition for the cellular nucleotide pool [16]. This allows the virus to better utilise cellular resources for its replication [1, 17, 18, 19, 20, 21]. In contrast, KSHV induces cell arrest in the G0/G1 phase through the transcription activator K‐Rta, which may be related to the early stages of viral reactivation [22]. Marek's Disease virus (MDV) and Varicella‐zoster virus (VZV), on the other hand, mediate the entry of cells into S phase, but the exact reason remains unclear [23, 24]. While viral‐induced cell cycle arrest and RNA Pol II hijacking are both strategies that enhance viral proliferation, the specific interplay between these two processes has not been well defined.

Although DNA replication and gene transcription of herpesviruses are two distinct biological processes in their life cycle, they are closely related due to the coupled nature of herpesvirus replication and transcription to maximise the efficiency of cellular resource utilisation [25, 26]. To achieve this, many herpesviruses have been found to establish a membrane‐less cellular compartments, called viral replication compartments (vRCs) [27, 28], which concentrate viral transcription and replication molecules [29, 30, 31], thereby spatially isolating the host's utilisation of cellular resources and blocking antiviral responses. In these vRCs, essential components such as viral transcription activation protein ICP4, single‐stranded binding protein ICP8 and host protein PML, etc. have been found playing crucial roles [17, 32, 33]. Therefore, the formation of vRCs is essential for both viral transcription and DNA replication.

Although considerable research has been conducted on the regulation of the host cell cycle by herpesviruses, the exact phases of the cell cycle targeted by different viruses vary. Given that the cell cycle regulation is closely tied to viral genomic DNA replication, we sought to explore whether the cell cycle itself contributes to viral gene transcription. To this end, we investigated the relationship and underlying mechanisms between cell cycle control and RNA pol II recruitment during an avian alpha‐herpesvirus (antid herpesvirus 1, AnHV‐1) infection. Our findings revealed that during AnHV‐1 infection, viral DNA synthesis arrests the cell cycle at the S phase, which facilitated the formation of vRCs and facilitates the preferential recruitment of RNA Pol II to transcribe viral genes. These findings offer new insights into the complex interactions between herpesviruses and host cell machinery, expanding our understanding of viral exploitation of host cellular processes.

## 2 Materials and Methods

### 2.1 Cells, Viruses and Drugs

Duck embryo fibroblast (DEF) cells were prepared from 9‐ to 11‐day‐old healthy duck embryos (Chengdu Kerimo Breeding Company, China) and cultured in Dulbecco's modified Eagle's medium (DMEM) supplemented with 10% newborn bovine serum (NBS) under 5% CO₂ at 37°C. AnHV‐1‐CHv (GenBank Accession No. JQ647509.1) or AnHV‐1‐BAC (Bacterial Artificial Chromosome) were generously provided by the Sichuan Agricultural University Avian Diseases Research Center. Phosphonoacetic acid (PAA) (4408‐78‐0, CHEJETER) (It was reported that the antiviral activity of PAA was specific to herpes viruses, while other DNA viruses (SV40) and human adenovirus Type 12 were not inhibited [34]) was dissolved in water at a concentration of 200 μg/mL and used for all drug treatments. S‐phase synchronisation was induced by adding 1 mM thymidine (HY‐N1150, MedChemExpress) to the culture medium for 24 h, while G2/M‐phase synchronisation was achieved by treating cells with 40 ng/mL nocodazole (HY‐13520, MedChemExpress) for 24 h.

### 2.2 Cell Viability Detection

The Cell Counting Kit‐8 (CCK‐8, BS350B, Biosharp) was employed to assess the effects of PAA on cell viability. DEF cells were seeded in 96‐well plates and cultured for 24 h, followed by PAA treatment. The cells were then incubated at 37°C for 20 min. The CCK‐8 assay was conducted according to the manufacturer's protocol, and absorbance was measured at 450 nm.

### 2.3 Viral Titer Detection Assay

DEF cells were seeded in 24‐well plates and infected with AnHV‐1 at a multiplicity of infection (MOI) of 1. Infected cells were incubated in medium with or without PAA. At the indicated time points, cells were collected by scraping into the medium and subjected to three freeze–thaw cycles to release cell‐associated virus. The viral titre of AnHV‐1 in DEF cells was determined using the median tissue culture infective dose (TCID₅₀) method. Following removal of the medium, 100 μL of 10‐fold serial dilutions of the virus were added to 96‐well plates. The cytopathic effect (CPE) was monitored every 24 h post‐infection (hpi) for 7 days. The viral titre was calculated using the Reed and Muench method.

### 2.4 Viral DNA Synthesis Detection

DEF cells were seeded in 24‐well plates and infected with AnHV‐1 at 1 MOI. Infected cells were incubated in medium with or without 200 μg/mL PAA. At 12/24/48/72 hpi, DNA was extracted using the HiPure Viral DNA Mini Kit (D3191‐03, Magen). Viral copy numbers were quantified by qPCR based on a standard curve generated from purified viral DNA. Primers specific for the UL30 gene were used for viral DNA amplification by the previously generated standard curve: *Y* = −4.262*X* + 43.675 [35].

### 2.5 Viral Fluorescent Plaques

DEF were seeded in a six‐well plates and infected with AnHV‐1 at an 0.01 MOI. Infected cells were incubated in medium with or without Thymidine. At 48/72/96 hpi, viral plaques were observed under a fluorescence microscope (ECLIPSE Ti‐S, Nikon).

### 2.6 RNA‐Seq

DEF cells were seeded in 24‐well plates and infected with AnHV‐1 at 1 MOI. Infected cells were incubated in medium with or without PAA. At 12 hpi, total RNA was isolated using the RNA‐easy Isolation Reagent (R701‐01, Vazyme) according to the manufacturer's recommendations. Total RNA was extracted using the mirVana miRNA Isolation Kit (Ambion) following the manufacturer's protocol. RNA integrity was evaluated using the Agilent 2100 Bioanalyzer (Agilent Technologies, Santa Clara, CA, USA). The samples with RNA Integrity Number (RIN) ≥ 7 were subjected to the subsequent analysis. The libraries were constructed using TruSeq Stranded mRNA LTSample Prep Kit (Illumina, San Diego, CA, USA) according to the manufacturer's instructions. Then these libraries were sequenced on the Illumina sequencing platform (HiSeqTM 2500 or Illumina HiSeq X Ten) and 125 bp/150 bp paired‐end reads were generated. The sequence reads were mapped to the duck genome (GCF_015476345.1) and duck enteritis virus (GCF_000885795.1) in the NCBI Reference Sequence database.

### 2.7 Real‐Time qPCR

Total RNA was extracted using the RNA‐easy Isolation Reagent (R701‐01, Vazyme) and reverse transcribed into cDNA using the Hifair II 1st Strand cDNA Synthesis SuperMix for qPCR with gDNA digester plus (11141ES60, Yeasen) according to the manufacturer's instructions. The relative mRNA expression levels of viral genes were quantified using the 2^−ΔΔCt method, normalised to cellular 18S RNA, and compared to control samples. Similarly, the relative expression levels of viral DNA were calculated using the 2^−ΔCt method and compared to those of the control group.

### 2.8 Western Blotting

DEF cells were seeded in 24‐well plates and infected with AnHV‐1 at 1 MOI. Infected cells were incubated in medium with or without PAA. At the indicated time points, proteins were extracted using lysis buffer, and Western blot analysis was performed. The following primary antibodies were used: Mouse anti‐ICP8 polyclonal antibody and rabbit anti‐gI polyclonal antibody (both produced in our laboratory), and mouse anti‐GAPDH antibody (60004–1, Proteintech). Secondary antibodies included HRP‐conjugated Affinipure Goat Anti‐Rabbit IgG (H+L) (SA00001‐2, Proteintech) and HRP‐conjugated Affinipure Goat Anti‐Mouse IgG (H+L) (SA00001‐1, Proteintech). Protein bands were visualised using an enhanced chemiluminescence (ECL) detection kit (Bio‐Rad), and band intensities were quantified with ImageJ.

### 2.9 Immunofluorescence Assay

DEF cells were grown on glass coverslips placed in 24‐well plates and infected with AnHV‐1 at an MOI of 1 or 10. Infected cells were incubated in medium with or without PAA. At the indicated time points, cells were washed with 1× PBS, fixed with 4% paraformaldehyde in PBS for 1 h at room temperature, and permeabilized with 0.5% Triton X‐100 in PBS for 20 min at room temperature. After washing three times with 1× PBS, cells were incubated with 5% BSA for 1 h at room temperature. Subsequently, cells were incubated overnight at 4°C with the following primary antibodies: anti‐ICP4 polyclonal antibody and anti‐ICP8 polyclonal antibody (both produced in our laboratory), Anti‐RNA pol II CTD (2629, CST), Anti‐RNA pol II CTD phospho‐S2 (ab5095, Abcam), and Anti‐RNA pol II CTD phospho‐S5 (13,523, CST). The slides were then washed three times with 1× PBS and incubated with secondary antibodies Alexa Fluor 568 goat anti‐mouse/rabbit IgG (A1104/A11011, Invitrogen) or Alexa Fluor 488 goat anti‐mouse/rabbit IgG (A32723/A1108, Invitrogen). Nuclei were stained with DAPI (C0060, Solarbio). Images were captured using a fluorescence microscope (ECLIPSE 80i, Nikon), and relative fluorescence intensities were quantified using ImageJ.

### 2.10 Cut&Tag

DEF cells were seeded in six‐well plates and infected with AnHV‐1 at 10 MOI. Infected cells were incubated in medium with or without PAA. At 9 hpi, 100,000 cells were harvested using trypsin digestion for further experiments. CUT&Tag assays were performed using the Hyperactive Universal CUT&Tag Assay Kit for Illumina (TD903‐01, Vazyme) according to the manufacturer's protocol. The primary antibody used was anti‐RNA pol II CTD (2629, CST), and the secondary antibody was the Unconjugated Secondary Antibody for CUT&Tag (Ab206‐01, Vazyme). DNA amplification was carried out using the TruePrep Index Kit V2 for Illumina (TD202, Vazyme), and amplified DNA libraries were purified with VAHTS DNA Clean Beads (N411, Vazyme). qPCR analysis of DNA was performed as previously described. The purified libraries were sequenced on the Illumina sequencing platform. Sequence reads were mapped to the duck genome (GCF_003850225.1) and duck enteritis virus genome (GCF_000885795.1) available in the NCBI Reference Sequence database.

### 2.11 Cell Cycle Assay

DEF cells were collected and fixed in 70% cold ethanol overnight at 4°C. After washing with PBS, the cells were stained with 0.5 mL of PI/RNase Staining Buffer (550,825, BD Biosciences) for 15 min at room temperature in the dark. Cell cycle distribution was analysed using an Accuri C6 flow cytometer (BD Biosciences), and the data were processed with ModFit LT 5.0.

### 2.12 Statistical Analysis

All statistical analyses were performed using GraphPad Prism (version 8.2.0). Data from cell experiments represent the results of at least three independent replicates. Statistical significance was assessed using two‐way analysis of variance (ANOVA) or *t*‐test, with significance levels indicated as follows: *p < 0.05, **p < 0.01, ***p <  0.001, **p <  0.0001.

## 3 Results

### 3.1 The Repression of AnHV‐1 DNA Replication Causes a Reduction in the Formation of vRCs

**FIGURE 1:**
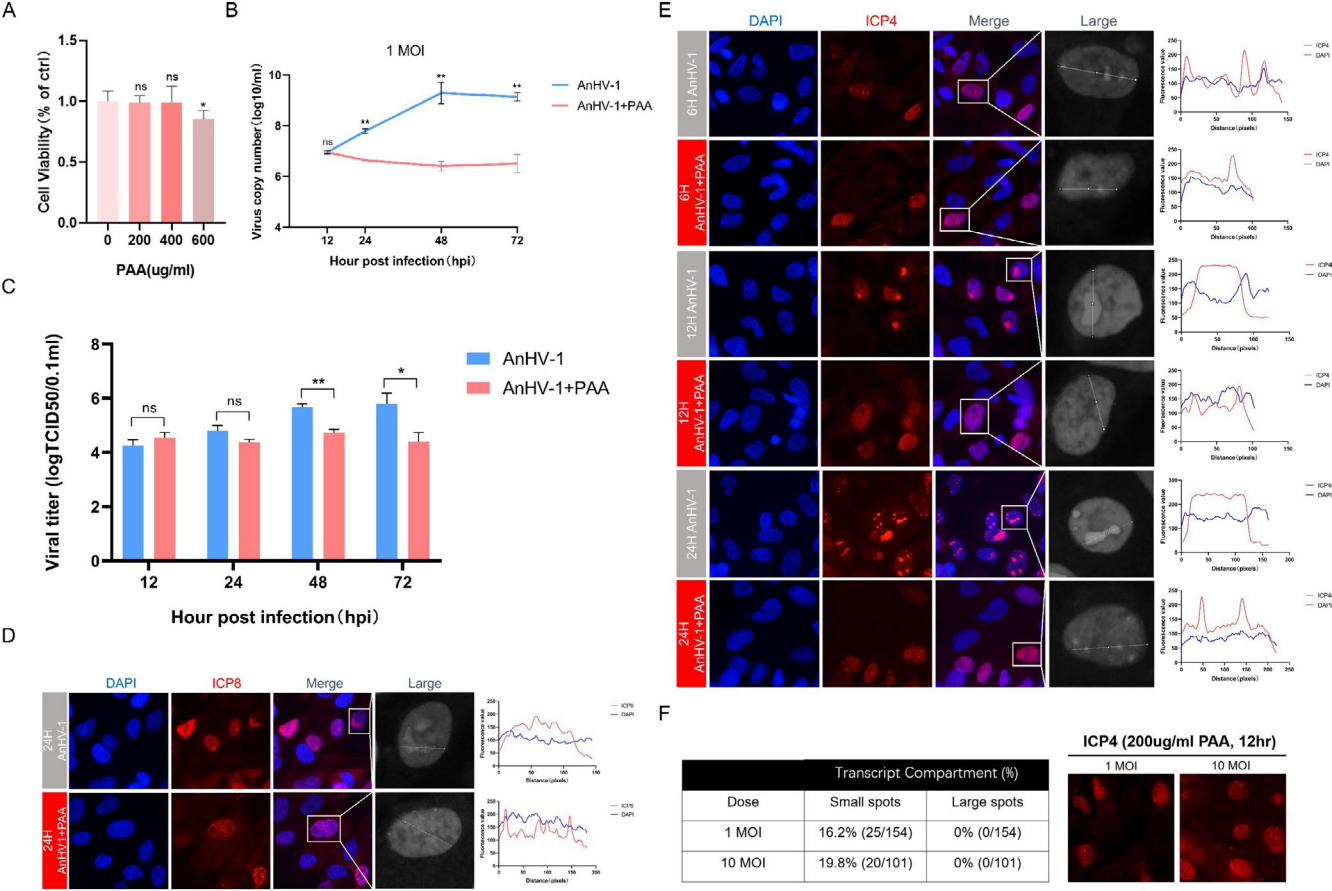
DNA replication of AnHV‐1 promotes the formation of viral replication factories. (A) DEF cells were treated with different doses of PAA (0–600 ug/ml) for 24 h. The cell viability was assessed by the CCK‐8 assay. (B, C) After infection with AnHV‐1 at 1 MOI and treated with PAA (200 ug/ml) or no drug, DEF cells were harvested at 12/24/48/72 hpi for titre and viral copy numbers analysis. The data were analysed by two‐way ANOVA. Each column was obtained from three independent experiments. (D, E) DEF cells were infected with AnHV‐1 at 1 MOI and treated with or without PAA (200 ug/ml), and on 6/12/24 hpi after infection fixed cells were tested and fixed cells were stained with ICP8 (red) (D) or ICP4 (red) (E) to examine the formation of vRCs. Each panel displays a magnified image of the transcribed site and shows the fluorescence intensity with a line graph. (F) DEF cells were infected 1 or 10 MOI AnHV‐1 and with PAA (200 ug/ml), cells were stained for ICP4 (red) to assess the formation of vRCs at 12 hpi. The number and morphological changes of ICP4‐positive cells were quantified manually. Images were captured at 40× magnification. *p < 0.05, **p < 0.01, ***p < 0.001, ****p < 0.0001, ns: Not significant.

vRCs are virus‐induced nuclear microenvironments that concentrate both viral and host factors to specific compartments, enabling the virus to hijack and control cellular processes to facilitate its replication. As mentioned earlier, ICP4 and ICP8 are key components within these vRCs. ICP4 plays a critical role in recruiting RNA Pol II to the viral genome, initiating the transcriptional cascade necessary for viral gene expression [32, 36, 37, 38]. The relocation of ICP8 from the pre‐replication sites to larger foci is tightly associated with vDNA synthesis and the formation of replication compartments, serving as a marker for the progression of vDNA replication [39, 40, 41, 42, 43]. Hence, both ICP4 and ICP8 are recognised as key markers of vRCs. Because both DNA synthesis and transcription of herpesviruses occur within vRCs, we initially explored the relationship between AnHV‐1 genome replication and the formation of vRCs. To do this, we employed PAA, a known herpesvirus DNA synthesis inhibitor, to block viral DNA replication [34], enabling us to observe the formation of vRCs and establish a model for inhibiting viral replication. First, we optimised the conditions for detecting the effect of PAA on DEF cell viability and determined that a concentration of at least 200 μg/mL PAA did not affect cell viability and effectively inhibited AnHV‐1 DNA synthesis during incubation (Figure 1A). By quantifying AnHV‐1 DNA copy numbers and virus titers following PAA treatment, we confirmed that PAA significantly inhibited DNA replication over the observation period, thereby reducing progeny virus production (Figure 1B,C). To examine the extent to which vRC formation relies on DNA synthesis, we performed IFA to visualise vRCs. The results showed that the addition of PAA led to the disappearance of large foci marked by ICP8 (Figure 1D). To further characterise the effect of DNA replication on the formation of the ICP4 transcription complex, which serves as a proxy for HSV‐1 vRCs [36], we categorised ICP4‐marked foci based on their size: Foci smaller than 2 μm were defined as “small spots” (indicating the absence of vRCs formation despite viral entry into the nucleus), while foci larger than 2 μm were defined as “large spots” (indicating successful vRCs formation). As a result, at the beginning of viral infection (6 hpi), ICP4 formed small spots and gradually gather together to large spots at 12 hpi, indicating significant growth changes in the foci between 6 and 12 hpi. At 24 hpi, the foci exhibited accelerated growth and irregular shapes (Figure 1E). However, the number of cells containing ICP4 foci varied according to the amount of vDNA after PAA treatment, and no large spots were formed, suggesting that the suppression of DNA replication affects the formation of vRCs instead of the number of vDNA (Figure 1F). These data indicate that efficient DNA replication is necessary to form and maintain vRCs. Inhibition of DNA replication can affect the maintenance and growth of vRCs.

### 3.2 The Inhibition of AnHV‐1 DNA Replication Leads to Reduced Recruitment of RNA pol II at RCs

**FIGURE 2:**
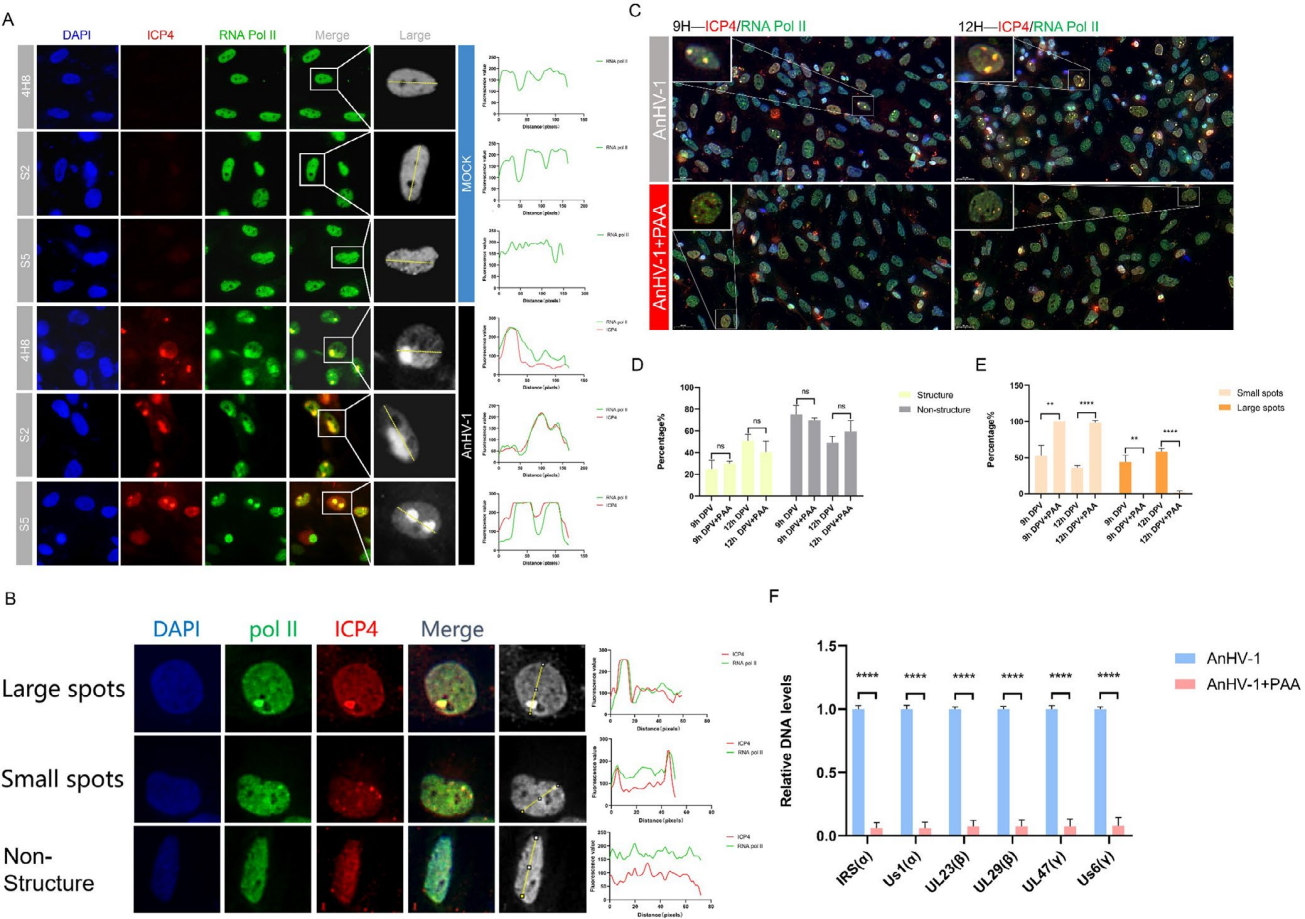
AnHV‐1 DNA replication was positively correlated with recruitment of RNA pol II to vRCs. (A) DEF cells were infected with AnHV‐1 at 1 MOI for 24 hpi. Fixed cells were stained for ICP4 (Red) and RNA Pol II (Green) to visualise the formation of replication compartments. Images were captured at 40× magnification. DEF cells were infected with AnHV‐1 10 MOI for 9/12 hpi and with or without PAA (200 ug/ml) in figure (B–E). (B) Quantification of morphological changes of viral replication foci. Each ICP4‐positive cell was classified into one of three categories: No structure, small spots (pre‐vRCs), or large spots (vRCs). (C) Images of replication factories are shown in each panel and arrows indicate magnified cell. (D, E) Manual cell counting was performed to quantify the morphological changes of vRCs induced by inhibiting vDNA replication. Under 40× magnification, three random fields were selected, and the number of ICP4‐positive cells co‐localised with RNA Pol II, along with associated morphological changes, was counted. (F) DEF cells were infected with AnHV‐1 at 10 MOI and treated with or without PAA (200 ug/ml). Cells were harvested 9 hpi for CUT&Tag assay. Changes in RNA pol II occupancy at each period gene were assessed by qPCR. The data were analysed by the *t*‐test. Each column was obtained from two independent experiments. *p < 0.05, **p < 0.01, ***p < 0.001, ****p < 0.0001, ns: Not significant.

To further investigate the relationship between vRCs and viral transcription, we examined the localisation of RNA pol II within vRCs. As shown in Figure 2A, following AnHV‐1 infection, total RNA pol II (4H8) and its phosphorylated states at serine 2 (S2) and serine 5 (S5) within the C‐terminal domain (CTD) formed clusters and co‐localised with ICP4 foci in the nucleus. Notably, Ser2 phosphorylation is closely associated with transcription elongation regulation, while Ser5 phosphorylation is primarily involved in transcription initiation [44, 45]. In the absence of viral infection, all three forms of RNA pol II were evenly distributed throughout the nucleus, suggesting that AnHV‐1 infection modulates distinct stages of transcription. Furthermore, the co‐localisation of RNA pol II with ICP4 in vRCs indicates that the redistribution of RNA pol II is directed toward supporting viral gene transcription. To further examine the role of viral DNA synthesis in the recruitment of RNA pol II to the viral genome, we used 4H8 as a representative marker in subsequent experiments. As shown in Figure 2B, we categorised the structures resulting from the co‐localisation of ICP4 and RNA pol II based on the criteria established in Figure 1E, with each ICP4 positive cell classified as either having no structure, Small spots (pre‐vRCs) or large spots (vRCs). Following AnHV‐1 infection, RNA pol II formed foci within the nucleus that were similar in size to ICP4 foci and co‐localized with them. Prior to PAA treatment, RNA pol II appeared as large foci that co‐localized with ICP4. However, after PAA treatment, both RNA pol II and ICP4 foci were reduced in size, though their co‐localisation was maintained (Figure 2C). To better understand the effect of PAA on RNA pol II recruitment during AnHV‐1 infection, we quantified RNA pol II‐ and ICP4‐positive nuclei, categorising them into structured and unstructured types. As shown in Figure 2D, 24.7% of RNA pol II formed foci that co‐localised with ICP4 at 9 hpi, increasing to 50.9% at 12 hpi. In contrast, after PAA treatment, this ratio was 30.1% at 9 hpi and 40.4% at 12 hpi. The number of cells containing significantly larger spots increased over time, with 44.2% of cells at 9 hpi and 57.9% at 12 hpi exhibiting such structures. However, following PAA treatment, large spots completely disappeared, with only one large spots observed at 12 hpi. Although the foci did not vanish entirely after replication inhibition, their size and number were significantly reduced compared to untreated conditions (Figure 2E).

To further confirm whether the reduction in RNA pol II localisation within vRCs results in decreased RNA pol II recruitment to the viral genome, we performed CUT&TAG‐qPCR to evaluate RNA pol II occupancy on representative viral genes. The results were consistent with those observed by IFA, showing a significant reduction in RNA pol II occupancy on viral genes in each period following PAA treatment. This finding suggests that the dispersal of replication factories impairs RNA pol II recruitment (Figure 2F). Additionally, analysis of previously obtained CUT&TAG sequencing data from our research group further confirmed that AnHV‐1 infection redistributes RNA pol II to the viral genome (Figure S1).

### 3.3 The Expression of Viral Genes Highly Depends on Effective Viral DNA Replication

**FIGURE 3:**
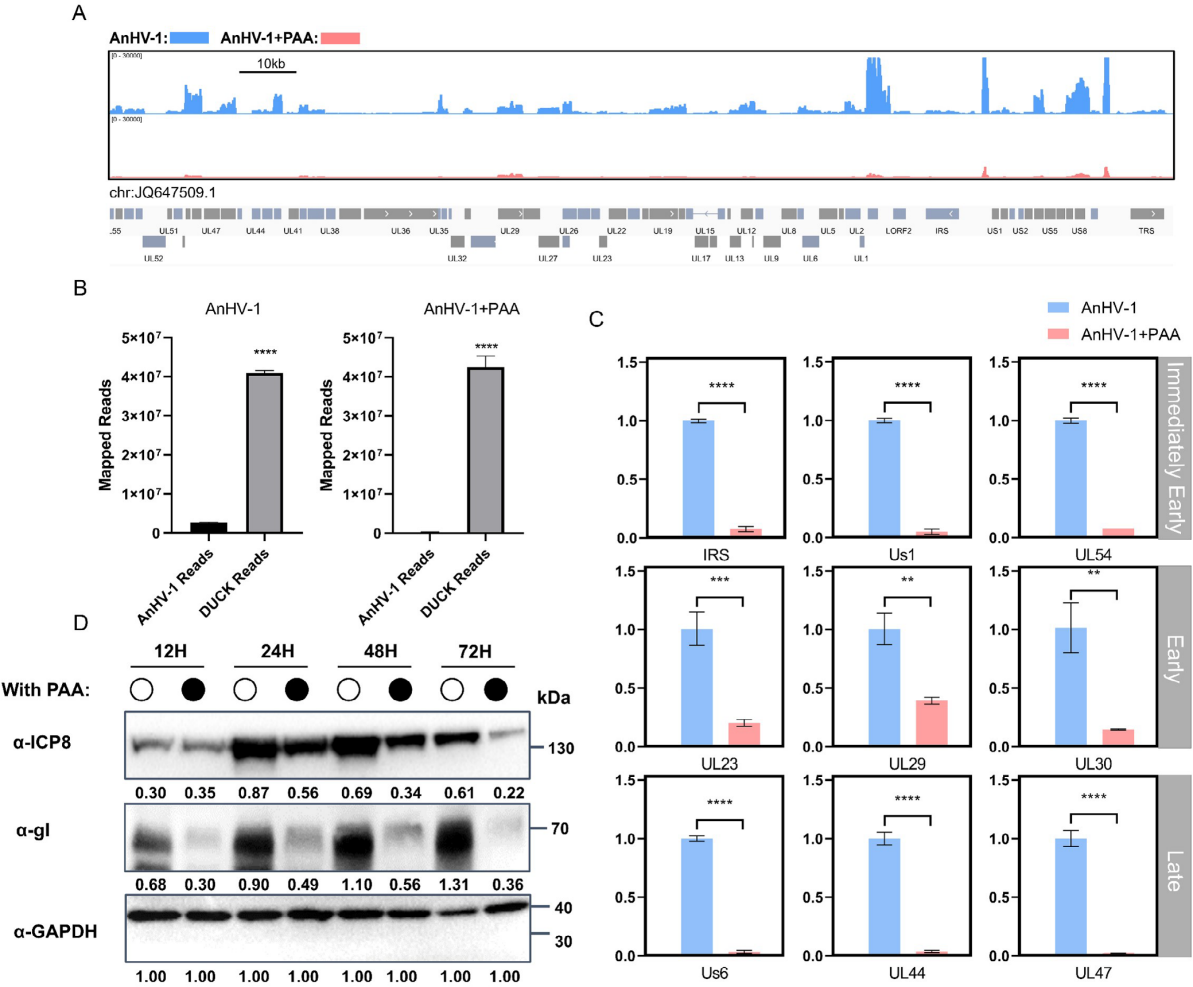
Suppression of AnHV‐1 genome replication extentsively decreased viral mRNA levels. (A) DEF cells were infected with AnHV‐1 at 1 MOI, with or without PAA (200 μg/mL) treatment. Samples were collected at 12 hpi for RNA‐Seq. Normalised read counts along the AnHV‐1 genome were displayed using the Integrative Genomics Viewer (IGV). The data shown in Figure 3A represent one representative replicate from each experimental group. Blue indicates the AnHV‐1 infection group, while red represents the group treated with PAA. (B) Distribution of RNA Pol II in duck and AnHV‐1 genomes in AnHV‐1 infection with PAA or without. (C) Determination the mRNA level of representvie genes at 12/24/48/72 hpi after with or without PAA treatment by RT‐qPCR. Each sample was repeated three times and the normalised gene is 18sRNA. (D) Changes in protein expression levels of ICP8 and gI were measured following drug treatment, normalised by GAPDH as an internal reference gene treatment. *p < 0.05, **p < 0.01, ***p < 0.001, ****p < 0.0001, ns: Not significant.

In the experiment described above, we observed that the recruitment of RNA polymerase II (RNA pol II) to ICP4 foci is dependent on the continuous replication of the viral genome. To evaluate the impact of reduced RNA pol II recruitment on viral genome transcription, we analysed the levels of viral mRNA using RNA‐Seq. In DEF cells treated with PAA, we found that mRNA levels across the entire AnHV‐1 genome were significantly reduced at 12 hpi compared to untreated controls (Figure 3A). Data presented in Figure 3B show that PAA treatment led to a substantial decrease in reads mapping to the viral genome at 12 hpi, with a drop from 5.9% to 0.66%, corresponding to an approximately 8.9‐fold reduction. Subsequent RT‐qPCR validation confirmed the RNA‐Seq findings, revealing that all tested viral genes were significantly suppressed following PAA treatment, with the most pronounced effects observed on late genes (Figure 3C). Furthermore, we expanded our analysis to include additional viral genes, and found that those involved in replication were also strongly repressed (Table S1). Western blot analysis indicated that the protein levels of ICP8, an early gene product, remained unchanged at 12 hpi in the presence or absence of PAA. However, by 24 hpi, ICP8 protein levels were reduced after PAA treatment. The expression of the late gene product gI was almost completely abolished following PAA treatment (Figure 3D). Collectively, these data suggest that inhibition of AnHV‐1 genome replication leads to a broad suppression of viral gene expression in each period, with the most severe effects on late genes.

### 3.4 AnHV‐1 Genome Replication Interfere With Host Gene Expression and Cell Progression

**FIGURE 4:**
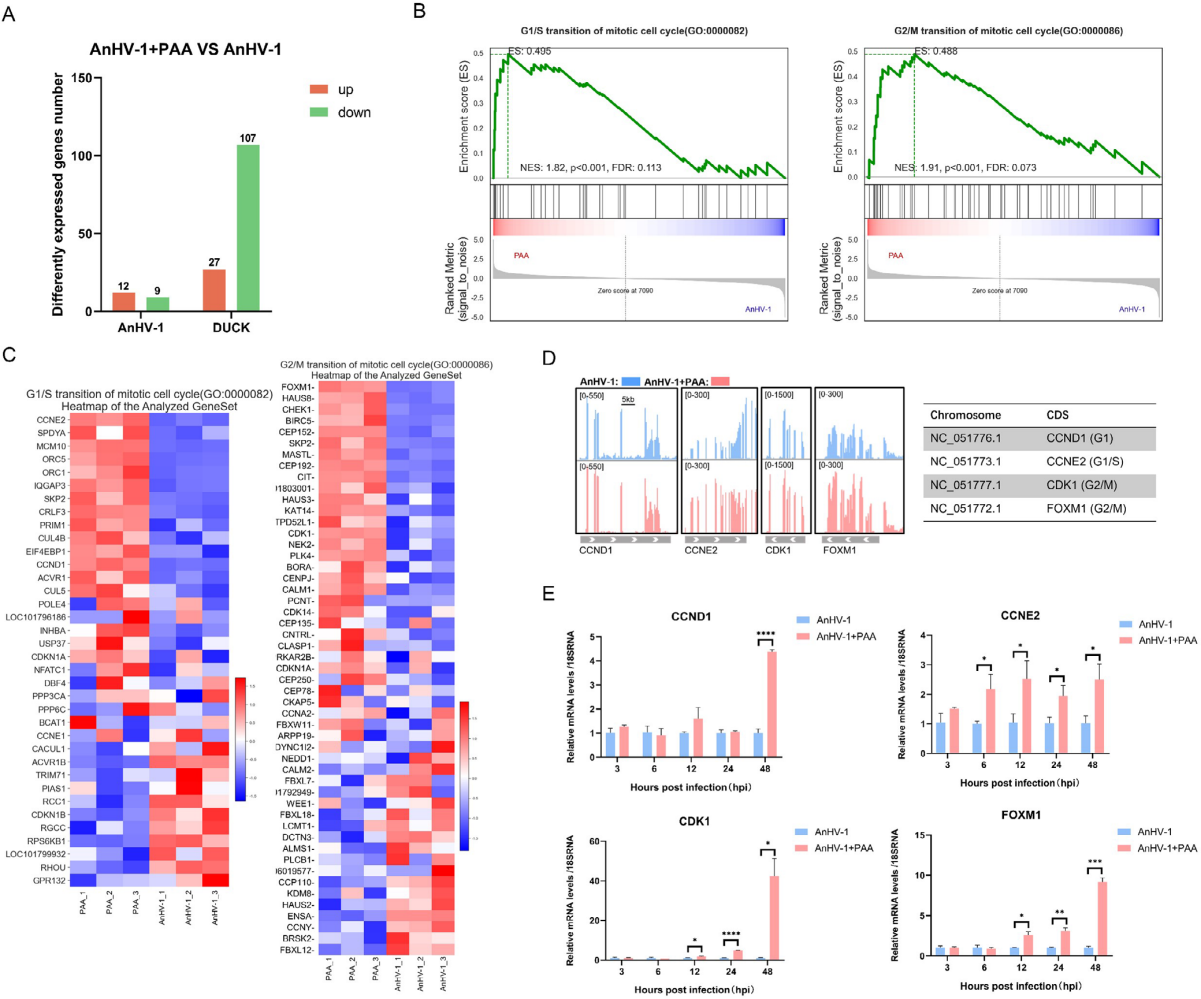
AnHV‐1 genome replication modulates host transcription and cell cycle progress. (A) *p* value < 0.05 and foldchange > 2 or foldchange < 0.5 was set as the threshold for significantly differential expression. where log2foldchange > 1 is differentially upregulated genes (indicated in red), log2foldchange < −1 is differentially downregulated genes (indicated in green). (B) The green line represents the enrichment score (ES) distribution of all genes. The position with the largest absolute value of the curve on the Y‐axis is the ES of the gene set. When ES > 0, the left side of the peak is the core gene, and ES < 0, the right side of the peak is the core gene. Vertical bars indicate the position of genes in the whole ranking of the gene set. Positive values are represented in red, negative values in blue, and values close to zero are shown in white. (C) The heatmap displays the expression levels of genes in each pathway, with red indicating relatively high expression and blue indicating relatively low expression. (D) The RNA‐Seq results are mapped to show peak changes in the host genome. (E) RT‐qPCR was used to detect changes in gene transcription levels at each infection stage at 3/6/12/24/48 hpi after drug treatment with three replicates of each sample, the normalised gene is 18SRNA. The data were analysed by two‐way ANOVA. Each column was obtained from three independent experiments. *p < 0.05, **p < 0.01, ***p < 0.001, ****p < 0.0001, ns: Not significant.

To investigate the underlying mechanism by which continuous viral DNA synthesis contributes to the recruitment of RNA pol II to vRCs and genomes, we analysed global gene expression changes in cells treated with PAA using RNA‐Seq. As shown in Figure 4A, a total of 27 upregulated and 107 downregulated host genes were identified following PAA treatment. Differentially expressed genes were further analysed using Gene Set Enrichment Analysis (GSEA). The results revealed that cell cycle‐related genes were prominently enriched at the top of the graph after PAA treatment, suggesting that PAA treatment rescued the downregulation of cell cycle‐related genes and pathways (G1/S phase and G2/M) that had been disrupted by AnHV‐1 infection (Figure 4B,C). Examination of normalised read counts along the duck genome using Integrative Genomics Viewer (IGV) revealed that key cell cycle genes were upregulated upon blocking viral replication (Figure 4D). RT‐qPCR validation of the RNA‐Seq results confirmed these findings (Figure 4E). Starting at 6 hpi, the mRNA level of the CCNE2 gene showed a significant increase, though the upregulation did not follow a clear temporal trend. In contrast, the expression of CCND1, CDK1, and FOXM1 in the PAA‐treated group increased progressively over time. These results suggest that AnHV‐1 genome replication may disrupt the normal cell cycle by modulating the expression of key cell cycle‐related genes.

### 3.5 AnHV‐1 Genome Replication Induced Cell Cycle S Phase Arrest to Promotes Viral Replication

**FIGURE 5:**
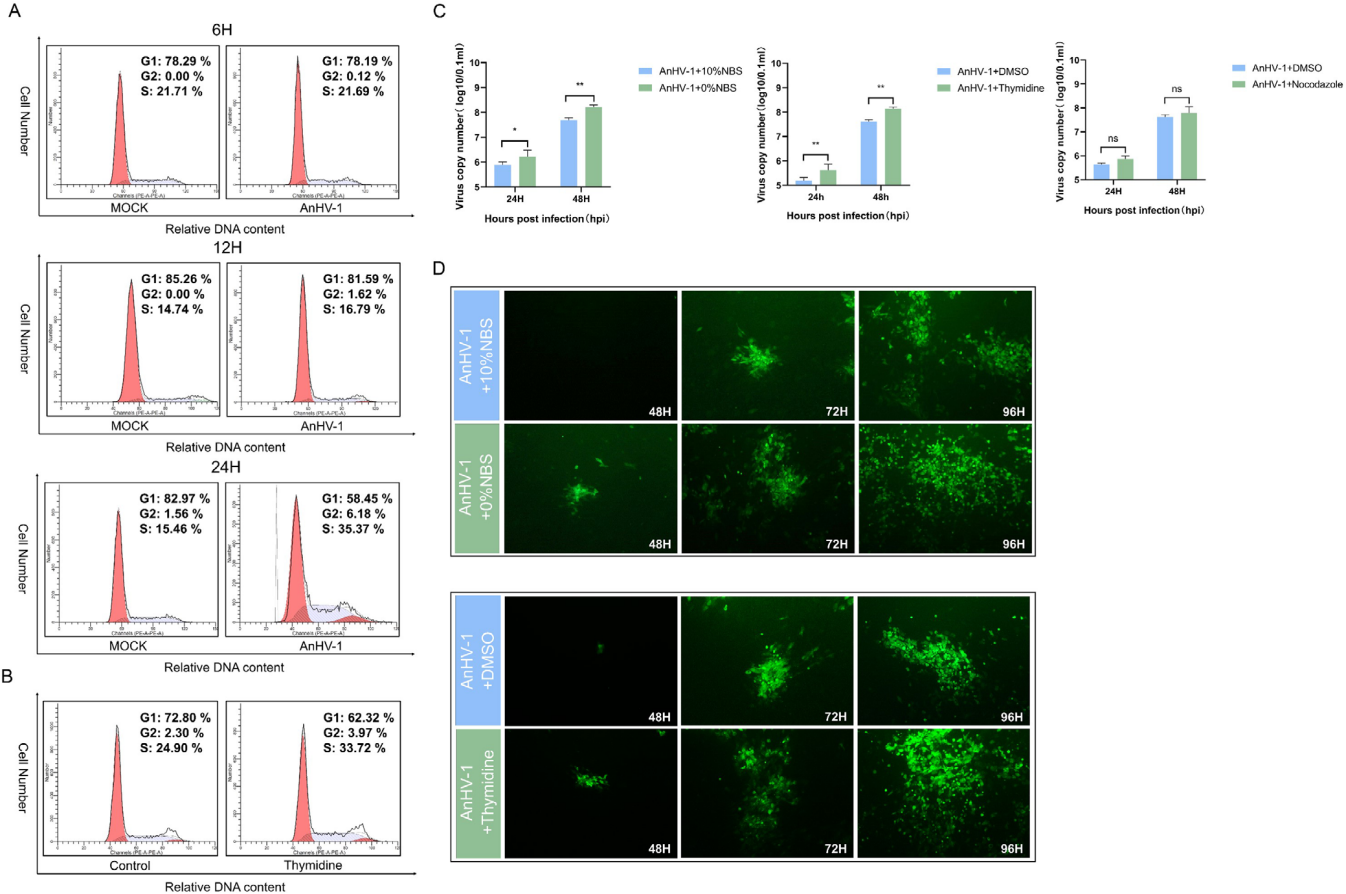
The cell cycle arrested at S phase contributes to AnHV‐1 replication. (A) DEF cells were infected with AnHV‐1 at 1MOI or not infected. The cell cycle distribution was analysed by flow cytometry at 6/12/24hpi. (B) DEF cells were treated with 1 mM thymine, and the cell cycle distribution was analysed by flow cytometry at 24 h. (C) DEF cells were treated in serum‐free medium, or with 1 mM thymine or 40 ng/mL nocodazole in complete medium. DNA was extracted at 24/48 hpi and viral copy numbers were quantified by qPCR. The data were analysed by two‐way ANOVA. Each column was obtained from three independent experiments. (D) DEF cells were treated with 1 mM thymidine or serum‐free medium for 24h, and then infected with 0.01 MOI AnHV‐1_BAC. Fields of view at magnification 20× at 48/72/96hpi. *p < 0.05, **p < 0.01, ***p < 0.001, ****p < 0.0001, ns: Not significant.

To test the hypothesis that AnHV‐1 genome synthesis controls the cell cycle, we performed flow cytometry to assess cell cycle changes following AnHV‐1 infection. The results indicated that at 6 hpi, AnHV‐1 had little effect on the cell cycle. However, from 12 hpi onward, the proportion of cells arrested in the S phase progressively increased. By 24 h, 35.37% of infected cells were arrested in the S phase, compared to only half that percentage in uninfected cells (Figure 5A). These data confirm that AnHV‐1 replication induces cell cycle arrest in the S phase in DEF cells. To examine the impact of S‐phase arrest on viral replication, we synchronised cells in the S phase using thymidine and observed results similar to those seen after AnHV‐1 infection (Figure 5B). Cells were treated with serum starvation, thymidine, or nocodazole before infection with AnHV‐1. The results showed a significant (5–10‐fold) increase in viral copy numbers in the serum starvation and thymidine‐treated groups compared to controls, while nocodazole treatment had no effect on viral DNA synthesis (Figure 5C). Additionally, the production of progeny virus was significantly higher in cells arrested in the S phase, as evidenced by an increase in fluorescent plaques of AnHV‐1. After serum removal at 48 hpi, the increase in AnHV‐1 fluorescence was less pronounced compared to the thymidine‐treated group (Figure 5D). Taken together, our data suggest that S‐phase arrest enhances AnHV‐1 replication, particularly during the early stages of infection.

### 3.6 Cell Cycle S Phase Arrest Promotes Viral Hijacking of Host RNA pol II

**FIGURE 6:**
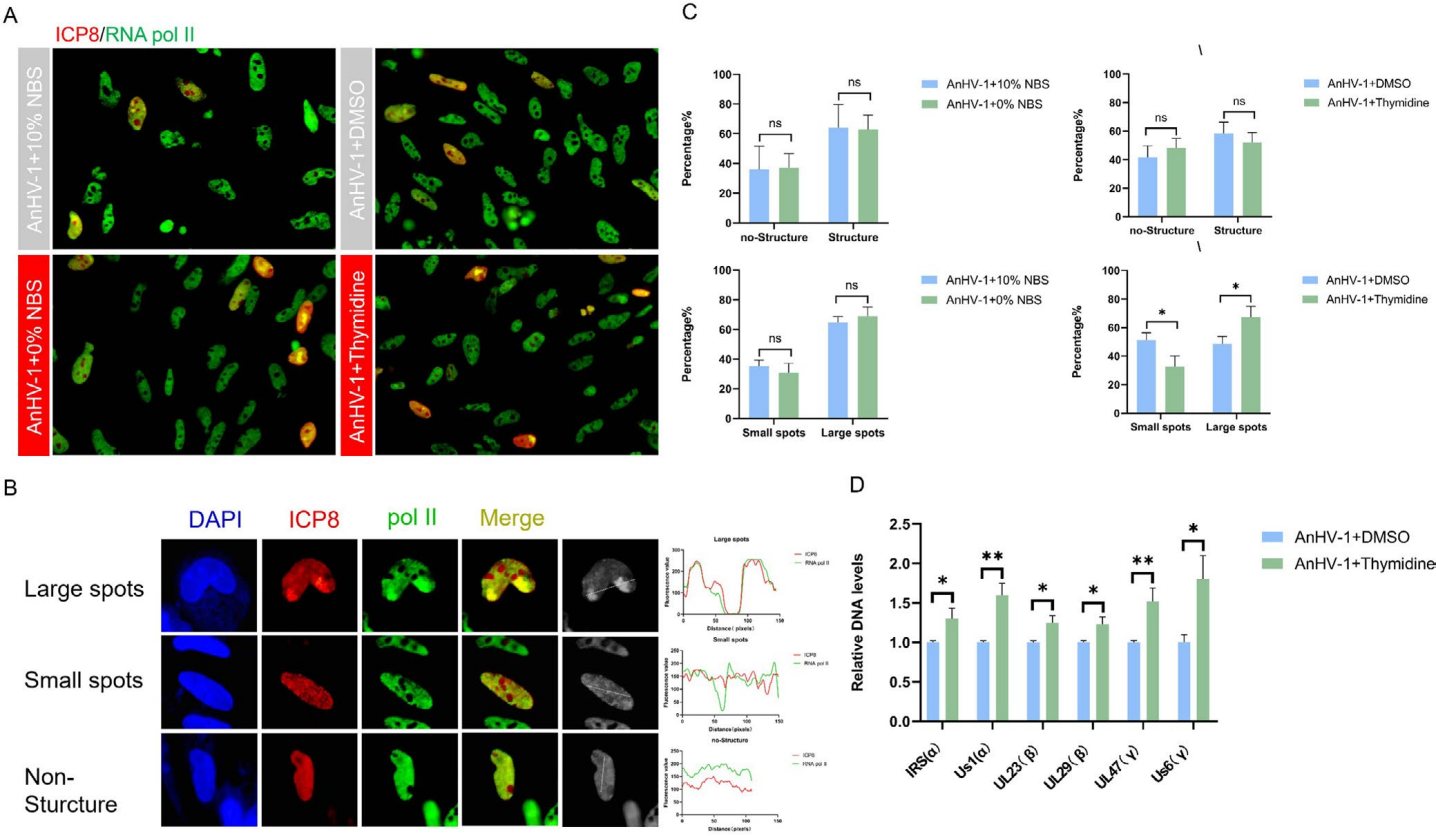
Arrest at the S phase of the cell cycle to promote AnHV‐1 hijacking RNA pol II. (A) DEF cells were inoculated with AnHV‐1 at 0.01 MOI, with or without PAA treatment (200 μg/mL). At 24 hpi, fixed cells were stained for ICP8 (red) and RNA pol II (green). Nuculei were classified as structureless, small foci, or large foci. (B) Images of morphological changes of viral replication foci are shown in each panel. The fields of view were captured at 40× magnification. (C) Manual cell counting method to quantify the number of co‐localization of ICP8 and RNA pol II and morphological changes in vRCs. (D) DEF cells were treated with 1 mM thymidine or no drug for 24h, and then infected with 0.01 MOI AnHV‐1. At 24 hpi, cells were harvested for CUT&Tag assay. Changes in RNA pol II occupancy at each period genes were detected by qPCR. The data were analysed by the *t*‐test. Each column was obtained from two independent experiments. *p < 0.05, **p < 0.01, ***p < 0.001, ****p < 0.0001, ns: Not significant.

To investigate the effect of cell cycle progression on AnHV‐1 recruitment of RNA pol II, cells were serum‐starved or treated with thymidine before AnHV‐1 infection to examine vRC formation and RNA pol II occupancy on the viral genome. The single‐stranded binding protein ICP8, which consistently co‐localises with the viral genome during replication, was used as a marker to assess RNA pol II recruitment to the viral genome. Initially, we quantified RNA pol II and ICP8‐positive nuclei and observed no significant effect of serum starvation on vRC formation. Under conditions of S‐phase arrest, we found that both RNA pol II and ICP8 formed three distinct structures: No structure, small spots, and large spots (Figure 6A). After thymidine treatment, the number of large spots significantly increased (Figure 6B), indicating a change in vRC formation. This increase in the number of vRCs suggests that thymidine treatment promotes viral genome replication. Furthermore, we observed enhanced recruitment of RNA pol II to these vRCs (Figure 6C). To further confirm the effect of S‐phase arrest on RNA pol II recruitment along the viral genome, we performed CUT&Tag qPCR analysis under thymidine treatment conditions. This analysis revealed a significant increase in RNA pol II occupancy on viral genes in each period following thymidine treatment (Figure 6D). These results indicate that the formation of vRCs and the recruitment of RNA pol II to the viral genome are enhanced when the cell cycle is arrested in the S phase.

## 4 Discussion

In recent years, the manipulation of the host cell cycle by herpesviruses has emerged as a crucial strategy for enhancing viral replication. Many herpesviruses arrest cells before the S‐phase to optimise the cellular environment for viral propagation [1]. For instance, EBV induce cells in the G1/S phase through the interaction between BORF2 and p53 [46]. This mechanism is also conserved in UL39, a homologue of HSV‐1. In addition, HSV‐1 also contains several viral proteins, such as ICP27 and ICP0, that block G1/S cycle progression [47, 48]. HCMV and murine cytomegalovirus (MCMV) also induce cells to the G1/S junction by upregulating CCNE1 and inhibiting CCNA2 expression [49, 50, 51, 52]. In contrast, AnHV‐1 arrests the cell cycle at the S‐phase, a strategy not commonly observed among other herpesviruses. This arrest facilitates viral DNA replication by providing a more favourable environment for the virus to hijack host resources during this critical phase of the cell cycle.

While much is known about viral manipulation of the cell cycle, the precise mechanisms and effector proteins driving AnHV‐1‐induced S‐phase arrest remain incompletely understood. However, it is plausible to speculate that other herpesviruses may block cells prior to the S phase to halt host gene expression, thereby generating a surplus of cellular resources that can be redirected to support viral replication. Given that AnHV‐1 arrests cells in the S phase, a stage in which host genome replication is already underway, a key question arises: How does AnHV‐1 compete for cellular resources to enhance its own replication? Our hypothesis is that DEF cells, upon entering the S phase, produce greater cellular resources and that AnHV‐1 takes advantage of this increased availability to secure more opportunities for replication. This may provide AnHV‐1 with a competitive advantage compared to cells in the G0 or G1 phases, despite the potential challenges posed by being in a more resource‐competitive phase.

In addition, AnHV‐1 may induce replication fork arrest or DNA damage during replication, resulting in the accumulation of excess nucleotides and other cellular materials that can be exploited by the virus. This hypothesis is supported by our finding that inhibition of viral genome replication significantly increases the transcript levels of CDK1 and CCNE2. CDK1 is a key protein involved in the DNA damage response and plays a critical role in the G2/M phase transition [53, 54]. Activation of the DNA damage response typically leads to the inhibition of CDK1 phosphorylation, thereby blocking cell cycle progression [55]. Furthermore, CCNE2 interacts with CDK2 to regulate both cell cycle progression and the DNA damage response [56, 57]. Based on these observations, we hypothesize that AnHV‐1 may induce cell cycle arrest through the induction of cellular damage. However, the specific viral proteins involved in this process and their precise roles remain to be further investigated.

Furthermore, cyclin‐dependent kinases (Cdks), which regulate the cell cycle, have been shown to directly activate the transcription of specific genes [58], and they also control the transcriptional cycle of RNA pol II [59]. However, the involvement of herpesviruses in this process has not been extensively reported. This gap in knowledge led us to focus on the impact of AnHV‐1 on cell cycle‐related genes, as demonstrated in our findings. While most research on herpesviruses has concentrated on how viral proteins interact directly with RNA pol II, our results showing that AnHV‐1 affects both CDK1 expression and RNA pol II recruitment have prompted us to hypothesize that AnHV‐1 may manipulate RNA pol II through a dual mechanism. Specifically, we propose that AnHV‐1 not only directly targets RNA pol II but also modulates its own gene transcription by indirectly influencing RNA pol II through alternative pathways. To explore this hypothesis, we used the cell cycle as a framework to investigate the interplay between AnHV‐1, the cell cycle, and RNA pol II. Our findings indicate that AnHV‐1 arrests cells in the S phase, which in turn facilitates the recruitment of RNA pol II. These results could provide new insights for developing novel antiviral drug targets. Currently, most antiviral drugs approved for the prevention and treatment of herpesvirus infections target the viral DNA polymerase. However, prolonged use of these antivirals has led to the emergence of drug resistance mutations within the DNA polymerase itself [60, 61]. Therefore, the development of alternative drug targets is crucial. Several CDK inhibitors are already in use in cancer research, and two inhibitors of Cdk7 are currently in clinical trials [62]. Targeting the cell cycle to create an environment unfavourable for viral replication may offer a promising strategy for developing new antiviral therapies. Additionally, given the pivotal role of RNA pol II in viral transcription, disrupting viral recruitment to RNA pol II, combined with cell cycle‐targeting strategies, may provide a potent approach to impair viral survival and enhance antiviral efficacy.

In our study, we also observed that the transcription of in each period genes was downregulated when viral genome replication was inhibited by PAA treatment. According to the cascade transcription model in herpesviruses, only late genes are typically regulated by viral genome replication. However, this observation can be explained by the widespread effects of inhibiting viral genome replication, as it impacts not only the transcription of late genes but also the production of progeny viruses [25, 26]. As a result, the expression of immediate‐early and early genes is suppressed, possibly due to a reduction in overall viral content. The data shown in Figure 1F indicate that the formation of AnHV‐1 vRCs is not dependent on the exposure dose but rather on genome replication. Increasing both PAA treatment and exposure dose by tenfold does not result in additional vRCs, suggesting that vRC formation depends on the viral DNA being in a functional state [63]. It has been previously demonstrated that vRCs formation requires continuous DNA replication, likely because the spatial configuration of newly replicated genome allows for the binding of replication complexes, RNA pol II, and other factors necessary for transcriptional activity [64, 65]. Based on these findings, we propose the following hypotheses regarding the role of S‐phase arrest in viral transcription: I. In the context of a continuously replicating viral genome, vDNA maintains an open conformation, creating an optimal environment for RNA pol II to enter the nucleus and bind to the promoter regions of viral genes. II. Arresting the cell cycle in the S phase disrupts the proper synthesis of double‐stranded DNA, impeding the transition to mitosis. If protein deposition occurs on the host genome, the efficiency of RNA pol II binding to the host DNA is reduced [66], which in turn makes more RNA pol II available for recruitment by the virus to its own genome.

Although our study provides valuable insights into the relationship between cell cycle arrest, viral replication, and RNA Pol II recruitment, there are several limitations to consider. First, our research focused on a single herpesvirus species, AnHV‐1, and a specific cell type (DEF cells). While our findings are important for understanding AnHV‐1's replication strategy, further studies on other herpesviruses and in different cell types are needed to generalise these results. Additionally, the precise molecular mechanisms underlying the S‐phase arrest induced by AnHV‐1 remain unclear. Future studies should aim to identify the viral proteins involved in this process and explore their interactions with host cell cycle regulators and DNA damage response factors.

In conclusion, our study reveals that AnHV‐1 arrests the host cell cycle in the S phase to optimise viral replication. This strategy, combined with the virus's ability to recruit RNA Pol II to the viral genome, provides a powerful mechanism for enhancing viral transcription and replication. Our findings also suggest that targeting the cell cycle and RNA Pol II recruitment could offer new avenues for antiviral therapy. Future research should aim to explore the molecular pathways involved in cell cycle arrest, viral genome replication, and RNA Pol II recruitment, as these processes are critical for understanding herpesvirus pathogenesis and developing effective antiviral strategies.

## Author Contributions

All listed authors made significant contributions to the completion of this article. Q.Q.Y. and Y.W. contributed equally to this study, with the author order reflecting their respective contributions. Specifically, Q.Q.Y. performed the experiments and drafted the manuscript, while Y.W. designed the study and revised the manuscript. M.S.W., S.C., R.Y.J., Q.Y., D.K.Z., M.F.L., X.X.Z., S.Q.Z., J.H., X.M.O., D.S., B.T., Y.H., and Z.W. participated in data analysis. A.C.C. provided critical revisions to the manuscript. All authors reviewed and approved the final version of the manuscript.

## Ethics Statement

No animal experiments were performed in this study.

## Conflicts of Interest

The authors declare no conflicts of interest.

## Supporting information


Data S1.

## Data Availability

Raw RNA‐Seq and CUT&Tag data are available in the Sequence Read Archive (SRA) public database under accession no. PRJNA1105761.
