# Supplementary material for: The S‐Phase Arrest of Host Cells Caused by an Alpha‐Herpesvirus Genome Replication Facilitates Viral Recruitment of RNA Polymerase II to Transcribe Viral Genes
Source: Cell Prolif. 2025 Jan 27;58(6):e13811. doi: 10.1111/cpr.13811 (PMC12179542; doi:10.1111/cpr.13811)
Supplement: Supplementary file 1 — Data S1. [file CPR-58-e13811-s001.docx]

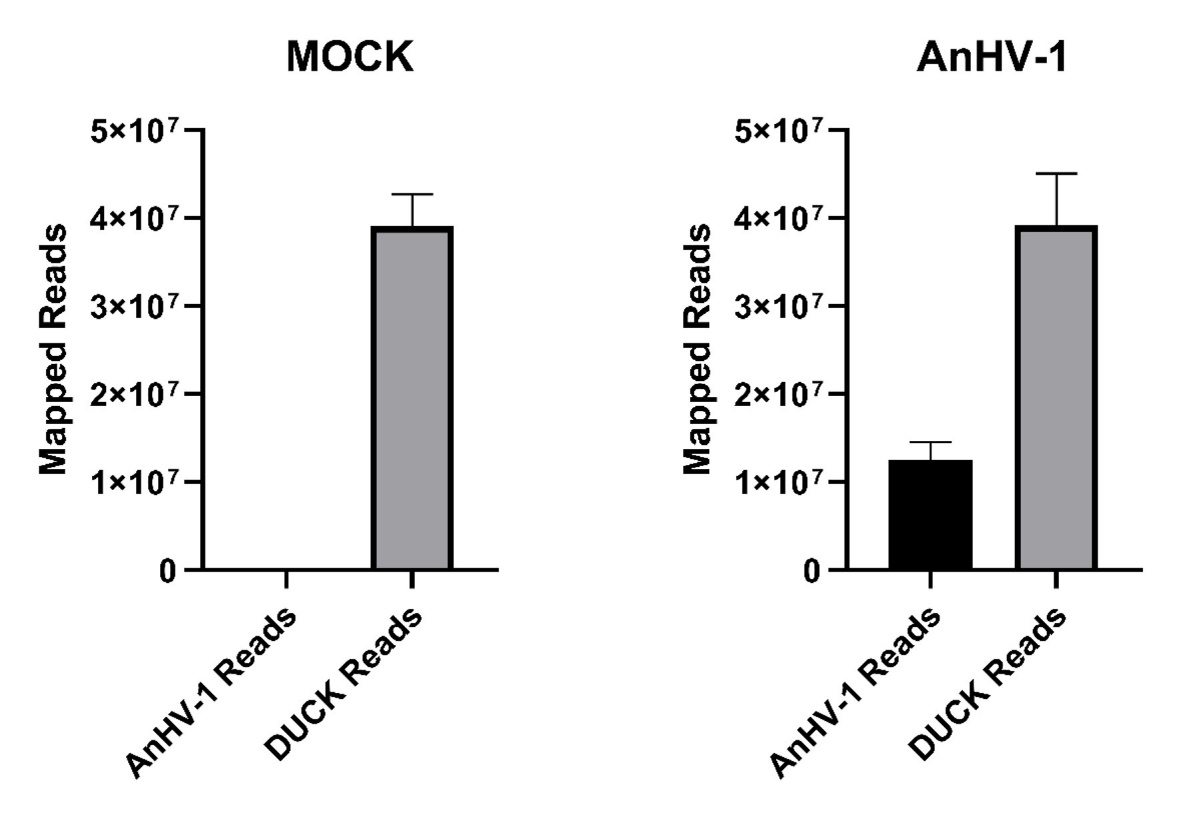


**Figure S1 Effect of AnHV-1 infection on the redistribution of RNA pol II in the virus genome**

AnHV-1 was inoculated into DEF cells at 10 MOI, and samples were collected for CUT&Tag-seq at 6 hpi. The changes in read counts within the AnHV-1 and host genomes were analyzed following AnHV-1 infection.


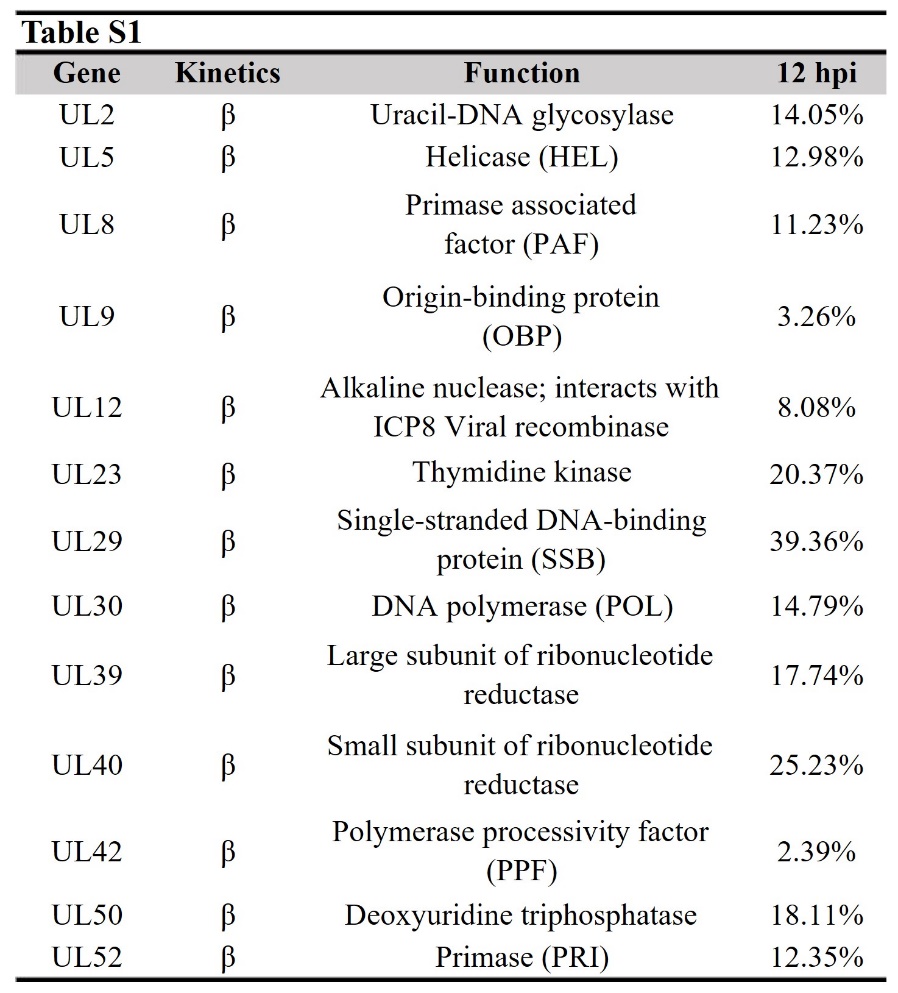


**Table S1 Suppression of AnHV-1 genome replication decreased transcription level of genes associated with replication**

DEF cells were infected with AnHV-1 at 1 MOI, with or without PAA (200ug/ml). The transcription levels of genes associated with replication were measured at 12 hpi following drug treatment, using RT-qPCR with three replicates of each sample. The normalized gene is 18sRNA
